# Hydroxyanthracene derivates citotoxicity: A differential evaluation between single molecule and whole plant extract

**DOI:** 10.3389/fpls.2023.1166075

**Published:** 2023-04-11

**Authors:** Laura Tinti, Vittoria Cicaloni, Paola Nezi, Giovanni Isoldi, Paolo Etiope, Barbara Barlozzini, Rita Pecorari, Laura Salvini

**Affiliations:** ^1^ Toscana Life Sciences Foundation, Siena, Italy; ^2^ Materia Medica Processing, Siena, Italy; ^3^ Linneus Consulting, Roma, Italy

**Keywords:** botanicals, hydroxyanthracene derivative, anthraquinones, phytochemical characterization, citotoxicity, proteomics and bioinformatics

## Abstract

Hydroxyanthracene derivates (HADs) are a group of natural or synthetic compounds with a wide range of biological activities (for instance, anti-inflammatory, antibacterial, and antiarthritic). In addition, because of their properties for helping the normal bowel function, HADs are widely used in constipation as pharmacological drugs and nutritional supplements. Nevertheless, during the past years, a safety usage of HAD products has been under consideration because some studies reported that HADs are not lacking toxicity (i.e., genotoxic and carcinogenic activity). Thus, the first objective of this study is to shed light on the large variability in composition of botanical food supplements containing HAD by a systematic analysis of the qualitative and quantitative composition of a cohort of extracts and raw materials of plants with high levels of anthraquinones commercially available (*Cassia angustifolia*, *Rhamnus purshiana*, *Rhamnus frangula*, *Rheum palmatum*, and *Rheum raponticum*). To date, the investigation of HAD toxicity was based on *in vitro* and *in vivo* studies conducted mainly on the use of the single molecules (emodin, aloe-emodin, and rhein) rather than on the whole plant extract. The qualitative-quantitative characterization was the starting point to select the most appropriate products to be used as treatment for our *in vitro* cell studies. Thus, the second objective of this study is the investigation, for the first time, of the toxic events of HAD used as single molecule in comparison with the whole plant extracts containing HAD in an intestinal *in vitro* model using human colorectal adenocarcinoma cells (Caco-2). In addition, a shotgun proteomics approach was applied to profile the differential protein expression in the Caco-2 cells after a single-HAD or whole–plant extract treatment to fully understand the potential targets and signaling pathways. In conclusion, the combination of a detailed phytochemical characterization of HAD products and a largely accurate analysis of the proteomic profile of intestinal cells treated with HAD products provided the opportunity to investigate their effects in the intestinal system.

## 1 Introduction

Hydroxyanthracene derivates (HADs) are a group of natural or synthetic compounds with broad applications including anti-inflammatory, antifungal, antibacterial, antiviral, and antiarthritic activity (Malik and Müller, 2016). In addition, because of their properties for supporting the regular bowel function, HADs are widely used in constipation as pharmacological drugs and nutritional supplements. However, because, in the literature, some papers reported that they are not lacking toxicity, the safety usage associated with HAD-containing products has been considered. In fact, some studies showed an increased risk of colorectal cancer for a prolonged use of product containing HAD (Cheng et al., 2020; Lombardi et al., 2020). Siegers et al. in 1993 demonstrated that chronic use of anthranoid laxatives, such as *Cassia angustifolia*, is considered to enhance the possibility to develop a colorectal cancer (Siegers et al., 1993). Moreover, in a colonic epithelial cells model, anthranoid laxatives have been shown to induce apoptosis within few hours after ingestion (van Gorkom et al., 2000). Animal studies also demonstrated increased toxicity after danthron administration (Walker et al., 1988).

Considering such studies, the European Food Safety Authority (EFSA) recently has re-evaluated the safety of the use of medicinal plants containing HAD in dietary supplements concluding that the HAD emodin, aloe-emodin, and the structurally related substance danthron have been shown to be genotoxic and carcinogenic until proven otherwise based on other studies. Furthermore, according to the current EU rules, the leaves of *Aloe vera* have been classified as possible carcinogenic substance (EFSA Panel on Food Additives and Nutrient Sources added to Food (ANS) et al., 2018; EMA and Committee on Herbal Medicinal Products (HMPC), 2019b; EMA and Committee on Herbal Medicinal Products (HMPC), 2019a 2019; European Commission Draft COMMISSION REGULATION (EU), 2020).

To the best of our knowledge, the data reported in the literature, concerning intestinal HAD toxicity, are mainly based on *in vitro* and *in vivo* studies evaluating the use of the single molecules (particularly emodin, aloe-emodin, and rhein), but there is no assessment of whole plant extract toxicity.

The large variability in composition of marketed products containing HAD currently on the botanical food supplements is well known, but few studies are reported about the qualitative and quantitative composition of these ones (Wang et al., 2012; Zhang et al., 2013; Loschi et al., 2022).

Therefore, phytochemical analysis of a representative number of herbal medicines, raw materials, and whole plant extracts was performed to obtain a qualitative-quantitative characterization of a large cohort of products containing HAD.

The analytical techniques largely used for the chemical characterization of HAD employ liquid chromatography (LC) coupled with UV detector. In this study, an analytical method was set up using LC coupled to a high-resolution mass spectrometer with the aim to explore the presence and the relative ratio between different anthraquinones, obtaining the profile of commercially available products with high levels of anthraquinones. Multivariate statistical analysis was additionally applied to understand the clustering and correlations between the investigated features and samples. This qualitative-quantitative characterization was then used to perform an accurate selection of the most appropriate products to be used as treatment for our *in vitro* cell studies.

The immortalized cell line of human colorectal adenocarcinoma cells (Caco-2) was employed as intestinal model to better understand the HAD toxic events between single molecule and the whole plant extracts (derived from *Cassia angustifolia*, *Rhamnus purshiana*, *Rhamnus frangula*, *Rheum palmatum*, and *Rheum raponticum*). First, cell viability was evaluated by the commonly used cytotoxicity assay that can elicit the preliminary HAD action, and then, an innovative shotgun proteomics approach was applied to profile the differential protein expression in the Caco-2 cells after a single-HAD or whole–plant extract treatment. Nowadays, proteomics coupled with bioinformatic analysis is increasingly used in biological research to fully understand the potential targets and signaling pathways. In our study, the proteomic approach was a powerful tool to perform high-throughput studies, allowing the investigation of proteome changes in response to performed treatment (single HAD versus whole plant extract).

The combination of a detailed phytochemical characterization of HAD products and a largely accurate analysis of the proteomic profile of intestinal cells treated with HAD products provided the opportunity to clarify their toxic events in the intestinal system.

## 2 Materials and methods

Twenty-nine samples were kindly supplied by different companies. Six samples of *Rhamnus Frangula*, five samples of *Rhamnus purshiana*, two samples of *Rheum palmatum*, one sample of *Rheum raponticum*, and 15 samples of *Cassia angustifolia* were analyzed. The samples were constituted by dried extract, bark, leaves, fruits, and vegetal material extracts. The list of the samples is summarized in **Table 1**.

**Table 1:** Samples analyzed and relative description.

Identifier	Description
C1	*Rhamnus purshiana* bark dried extract 10% cascarosides
F1	*Rhamnus frangula* bark dried extract ≥ 7.5%–9% glucofrangulines
S1	*Cassia angustifolia* leaves extract 6%–8% sennosides
R1	*Rheum palmatum* root powder (vegetal matrix)
F2	*Rhamnus frangula* bark (vegetal matrix)
S2	*Cassia angustifolia* leaves powder (vegetal matrix)
S3	*Cassia angustifolia* dried extract 20% sennosides
C2	*Rhamnus purshiana* dried extract
F3	*Rhamnus frangula* bark (vegetal matrix)
S4	*Cassia angustifolia* leaves dried extract ≥ 20% sennosides
S5	*Cassia angustifolia* leaves extract 6%–8% sennosides
F4	*Rhamnus frangula* dried extract from bark ≥ 7.5%–9% glucofrangulines
C3	*Rhamnus purshiana* dried extract 10%–12% cascarosides
F5	*Rhamnus frangula* bark (vegetal matrix)
C4	*Rhamnus purshiana* bark (vegetal matrix)
S6	*Cassia angustifolia* leaves dried extract ≥ 20% sennosides
S7	*Cassia angustifolia* dried extract 6%–8% sennosides
S8	*Cassia angustifolia* dried extract 20%
S9	*Cassia angustifolia* bulk sample
S10	*Cassia angustifolia* dried extract 6% sennosides
S11	*Cassia angustifolia* dried extract 20% sennosides
S12	*Cassia angustifolia* 6% sennosides
C5	*Rhamnus purshiana* bark powder (vegetal matrix)
F6	*Rhamnus frangula* bark powder (vegetal matrix)
R2	*Rheum raponticum* rhizome powder (vegetal matrix)
S13	*Cassia angustifolia* leaves powder (vegetal matrix)
R3	*Rheum palmatum* rhizome powder (vegetal matrix)
S14	*Cassia angustifolia* fruits powder (vegetal matrix)
S15	*Cassia angustifolia* leaves dried extract 12% sennosides

The reference standard compounds aloin A and B, aloe-emodin, emodin, cascaroside A, sennoside A and B, frangulin A and B, glucofrangulin A and B, rhein, physcion, chrysophanol, and rhein-8-glucoside were purchased from Extrasynthese. Danthron was purchased from PhytoLab. LC-MS–grade acetonitrile, formic acid, methanol, and ultrapure water were purchased from Merck Group.

### 2.1 Preparation of standard solution and samples

Standard stock solutions were prepared by dissolving 10 mg of each compound in 1 ml of dimethyl sulfoxide (DMSO; Merck Group). Working standard solutions were prepared by diluting aliquots in 60% methanol of each stock solution to obtain eight calibration mixtures in the range of 0.01–20 ppm of each analyte.

Solid matrix samples were prepared as follows: 50 mg of powder was dissolved in 10 ml of 60% methanol, ultrasonicated for 20 min, and centrifugated (13,000 rpm, 4°C) for 10 min. The supernatants were transferred in clean vials. Because the analytes of interests for this work showed a wide dynamic range of concentrations, the samples prepared as abovementioned were analyzed both without any further manipulations and diluted 10, 100, 200, 500, and 1000 times. For each sample, six analyses were performed in duplicate.

### 2.2 Quantification of HAD

The quantification of the HAD analytes in the extracts was performed using an Ultimate 3000 UPLC system (ThermoFisher Scientific) coupled with a high-resolution Q-Exactive Plus Hybrid Quadrupole-Orbitrap™ mass spectrometer (ThermoFisher Scientific).

The mass spectra were acquired in electrospray ionization in a negative ion mode. The targeted analysis was performed by parallel reaction monitoring (PRM) scan. In PRM, a list of target compounds could be monitored with high specificity: in fact, both the “targeted” analytes and their fragment ions were acquired in high-resolution mode, helping in differentiating background from the target ions.

The fragmentation mass spectrum (MS/MS) parameters were optimized for each analyte by direct infusion of a solution of each reference standard. Successively, the gradient was optimized by first injecting each reference compound and then using a mixture of all the analytes.

MS/MS spectra were recorded using the following parameters: spray voltage of 3.0 kV (negative), sheath gas of 20 (arbitrary units), auxiliary gas of 5.0 (arbitrary units), capillary temperature of 320°C, and resolution of 35,000. MS/MS spectra were obtained by a Higher Energy Collision Dissociation (HCD) of 30 (arbitrary units).

The final experimental conditions were reported in the following paragraph. The samples were analyzed in gradient using a column Acquity UPLC BEH C18 (2.1 mm × 15 cm, 1.7 µm, Waters). The mobile phases consisted of solvent A (0.1% formic acid in water) and solvent B (0.1% formic acid in acetonitrile). The gradient started with 15% of B, which was increased to 20% in 1 min. Then, the organic phase was increased up to 35% in 7 min. At 14 min, the phase B was raised to 90% and hold for 6 min before restoring the initial conditions. The flow rate was maintained at 0.200 ml/min, and the injection volume was 20 µl. In addition, the column temperature was kept at 35°C.

Each analyte was uniquely recognized by considering the retention time and the MS/MS spectrum.

For the quantification, a calibration curve of each analyte of interest was recorded before and after running the samples. Concentrations starting from 0.01 to 20 ppm were considered. For each analyte, a characteristic fragment ion from the MS/MS mass spectrum was chosen for the quantification (a detailed description was reported in Results and Discussion).

### 2.3 Statistical analysis

The data resulting from the abovementioned semi-quantitative analysis were imported into MetaboAnalyst 5.0 online platform (Chong et al., 2019).

The obtained matrix was normalized, and a log transformation (base 10) was performed. An heatmap was plotted to visualize the variations in samples and to separate metabolites into different subgroups by a hierarchical cluster analysis (HCA) based on Pearson distance. Successively, supervised multivariate modeling approaches such as partial least-squares discriminant analysis (PLS-DA) were applied to determine where the greatest variation lies in the data and to reveal the similarities or differences among the metabolite profiles relative to the different sample groups. The importance of each variable in the projection used in the PLS-DA model was represented by a variable importance in projection (VIP) score. VIP is a quantitative estimation of the discriminatory power of each individual feature. Variables with a VIP score of ≥1 was considered important in the PLS-DA model, indicating components that play a role in samples differentiation. Only components with VIP > 1.0 and p < 0.05 were selected as potential markers.

### 2.4 Cell viability assay

For viability assay, Caco-2 cell lineages were cultured in 24 well plates (5 × 10^4^ cells per well) in DMEM (Life Technologies) supplemented with 10% fetal bovine serum (FBS) and antibiotics (Life Technologies) at 37°C and 5% CO_2_. After 24 h, the cells have undergone a starvation step replacing the medium with fresh medium supplemented with 2% FBS. After 3 h, the cells were exposed to different concentrations (1–20 ppm) of the single compounds (emodin, rhein, and aloe-emodin) and the crude extracts of different plants (*Cassia angustifolia*, *Rhamnus purshiana*, *Rhamnus frangula*, and *Rheum palmatum*) containing the same concentrations (1–20 ppm) of the abovementioned analytes. The Caco-2 treatment was performed for 48 h. Then, the oxidized alamarBlue reagent (Merck Group) was added in each well at a final concentration of 0.15 mg/ml, and cells were maintained for 3 h in the standard culture conditions. The fluorescence from reduced alamarBlue reagent was measured using a microplate reader at 560-nm emission and-590 nm excitation wavelengths (Bonnier et al., 2015). The results were indicated as the percentage of viable cells in relation to the control of each compound. A negative control, containing the medium with no cells to determine baseline signal, was included in all assays. A T-test was used and results with p < 0.05 were considered statistically significant. Each abovementioned experimental condition was performed in triplicates.

### 2.5 Proteomics analysis

To evaluate the overview of proteins expressed after the anthraquinone treatment as single molecule or whole plant extract in Caco-2 cells, the LC-MS/MS analysis was executed, and a bioinformatic elaboration of detected proteins was performed with particular focus on apoptotic processes. Briefly, filter-aided sample preparation (Wiśniewski et al., 2009) was used for proteomic analysis of cell lysates. Cells were lysed in cold cell radioimmunoprecipitation buffer (ThermoFisher Scientific). The protein concentration was determined using BCA assay procedure (ThermoFisher Scientific). In particular, 200 μg of proteins were combined with 8 M urea to a final volume of 400 μl, uploaded in centrifugal ultrafiltration units with 30-kDa nominal molecular weight cutoff (Microcon ^®^ -30, Sigma-Aldrich). Successively, to reduce protein disulfide bridges, samples were incubated at Room Temperature (RT) for 30 min with 40 μl of 100 mM dithiothreitol and then centrifuged at 13,800 rpm for 30 min. Once the flowthrough was discarded, the filter was then washed twice using 400 μl of 8 M urea by 30 min of centrifugation at 13,800× g rpm. After that, the samples were incubated for 30 min in the dark to alkylate-free thiol groups with 100 μl of 100 mM iodoacetamide in 8 M urea. Filter units were washed first with 400 μl of 8 M urea and, successively, with 400 μ, of 50 mM ammonium bicarbonate, by centrifuging at 13,800× g rpm for 30 and 20 min, respectively. Proteins digestion was carried out at 37°C overnight using 1:50 dilution of Trypsin Gold-Mass Spec Grade (Promega) in 50 mM ammonium bicarbonate. After that, a centrifugation at 13,800× g rpm for 10 min was carried out to collect peptides, followed by two washes with 100 μl of 0.1% Formic Acid (FA) in distilled water. The digested samples were then desalted by using OASIS cartridges (Waters), brought to dryness, and reconstituted in formic acid (0.1%) in water to have a final concentration of 1 μg/ml. Q Exactive™ HF-X hybrid quadrupole-Orbitrap™ mass spectrometer (ThermoFisher Scientific) was used to perform LC-MS/MS analyses. The peptide separation was carried out at 35°C using a PepMap TM RSLC C18 column, 75 μm × 150 mm, 2 μm, 100 Å (ThermoFisher Scientific) at a flow rate of 300 nl/min. The mobile phases A and B used for the analysis were 0.1% formic acid in water and 0.1% formic acid in 80% acetonitrile, respectively. The gradient started with 5% of B, that was maintained constant for 5 min. Then, the organic phase was increased up to 90% in 97 min and kept constant for 9 min and then returned to the initial conditions. The mass spectrometer was set up in a data-dependent acquisition mode in which the most intense ions, a maximum of 12 in this kind of experiments, from a full MS scan spectrum (200–2,000 *m/z*) were selected for fragmentation.

Protein identification was performed using Proteome Discover 2.5 (ThermoFisher Scientific) and Sequest algorithm. The reference database was *Homo sapiens* (Taxonomy ID: 9606), and the number of total proteins was 204,961, downloaded in June 2022 from UniProtKB.

## 3 Results

### 3.1 Phytochemical samples characterization

**Figure 1:**
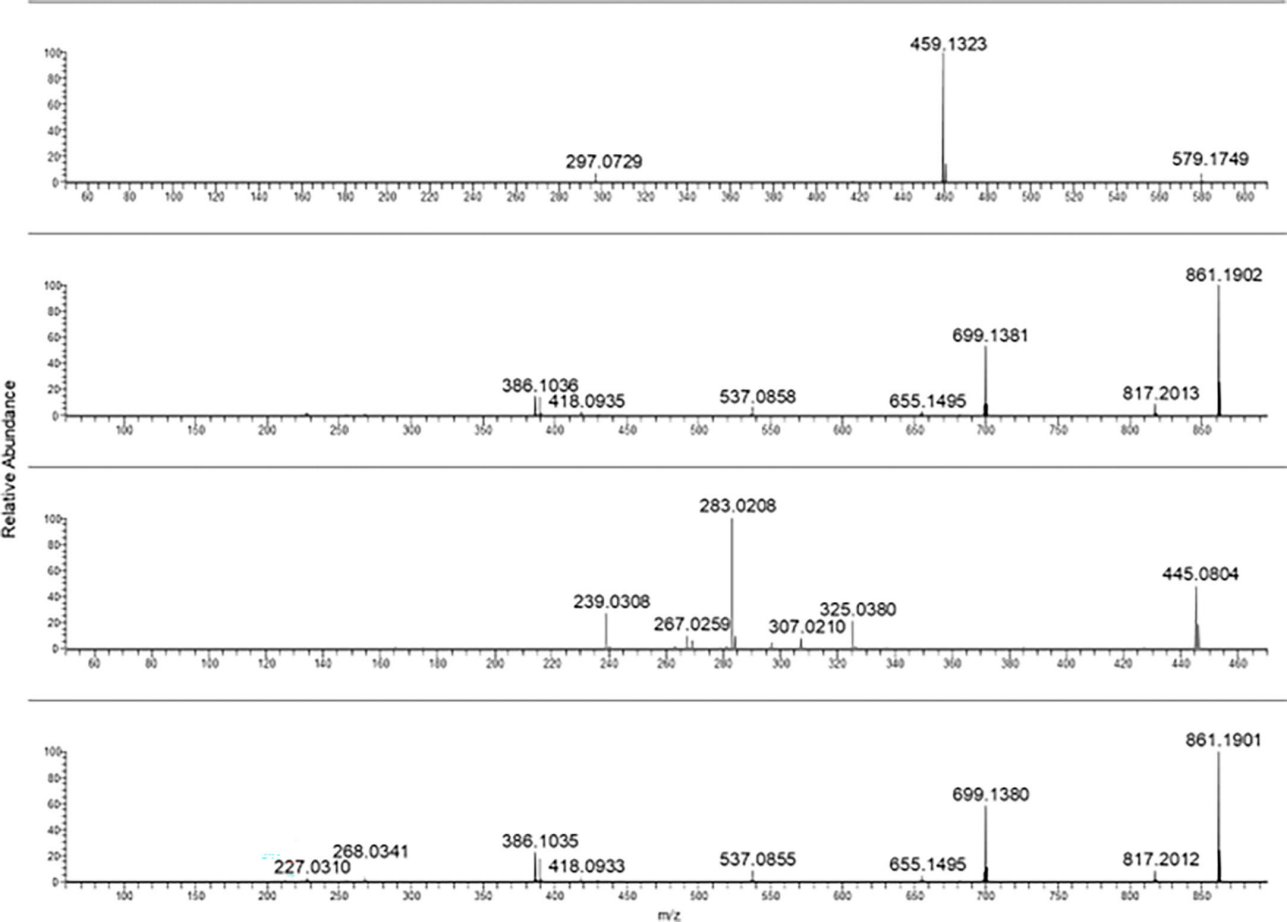
From top to bottom: cascaroside A, sennoside B, rhein-8-glucoside, and sennoside A MS/MS spectra.

**Figure 2:**
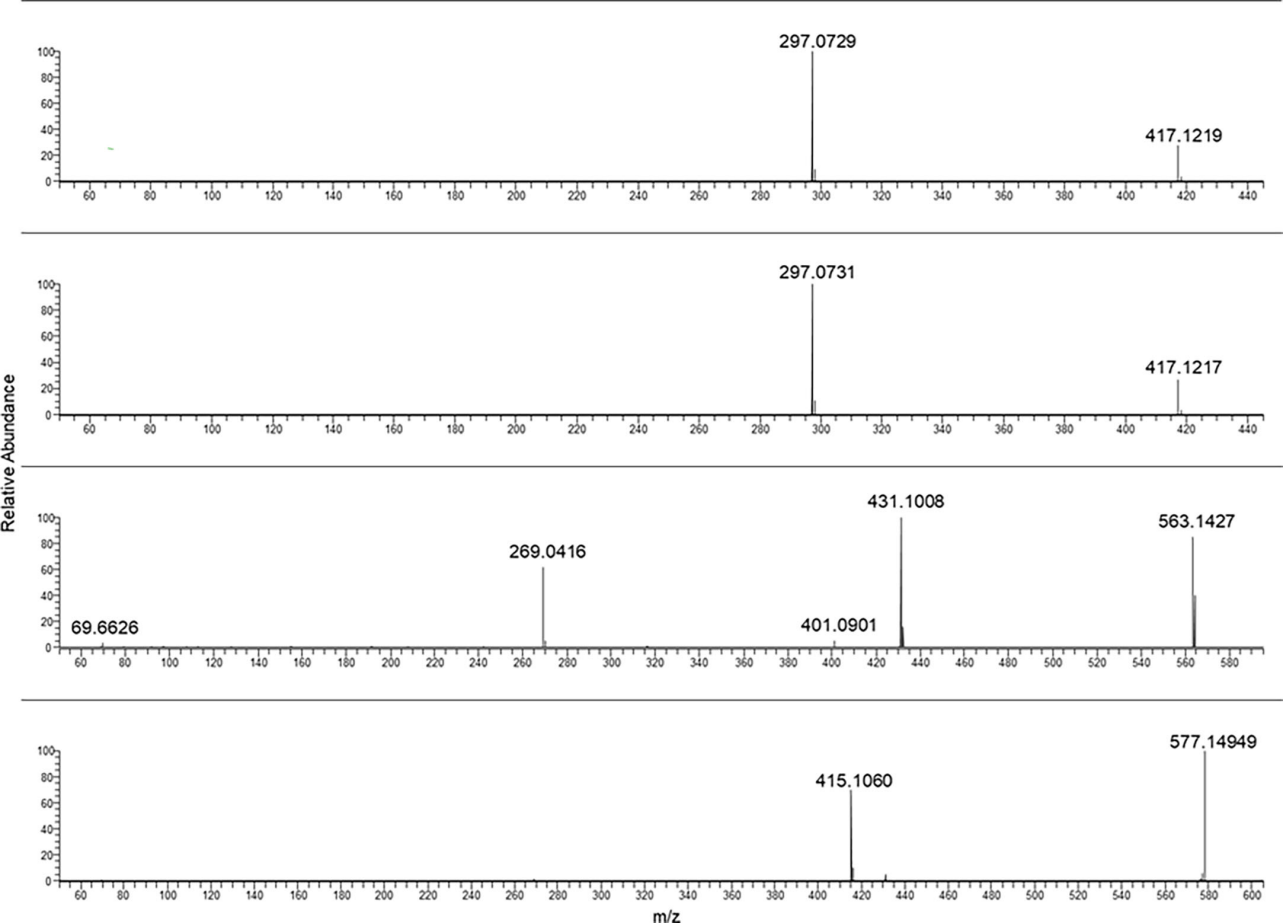
From the top to bottom: Aloin B aloin A glucofrangulin B and glucofrangulin A MS/MS spectra.

**Figure 3:**
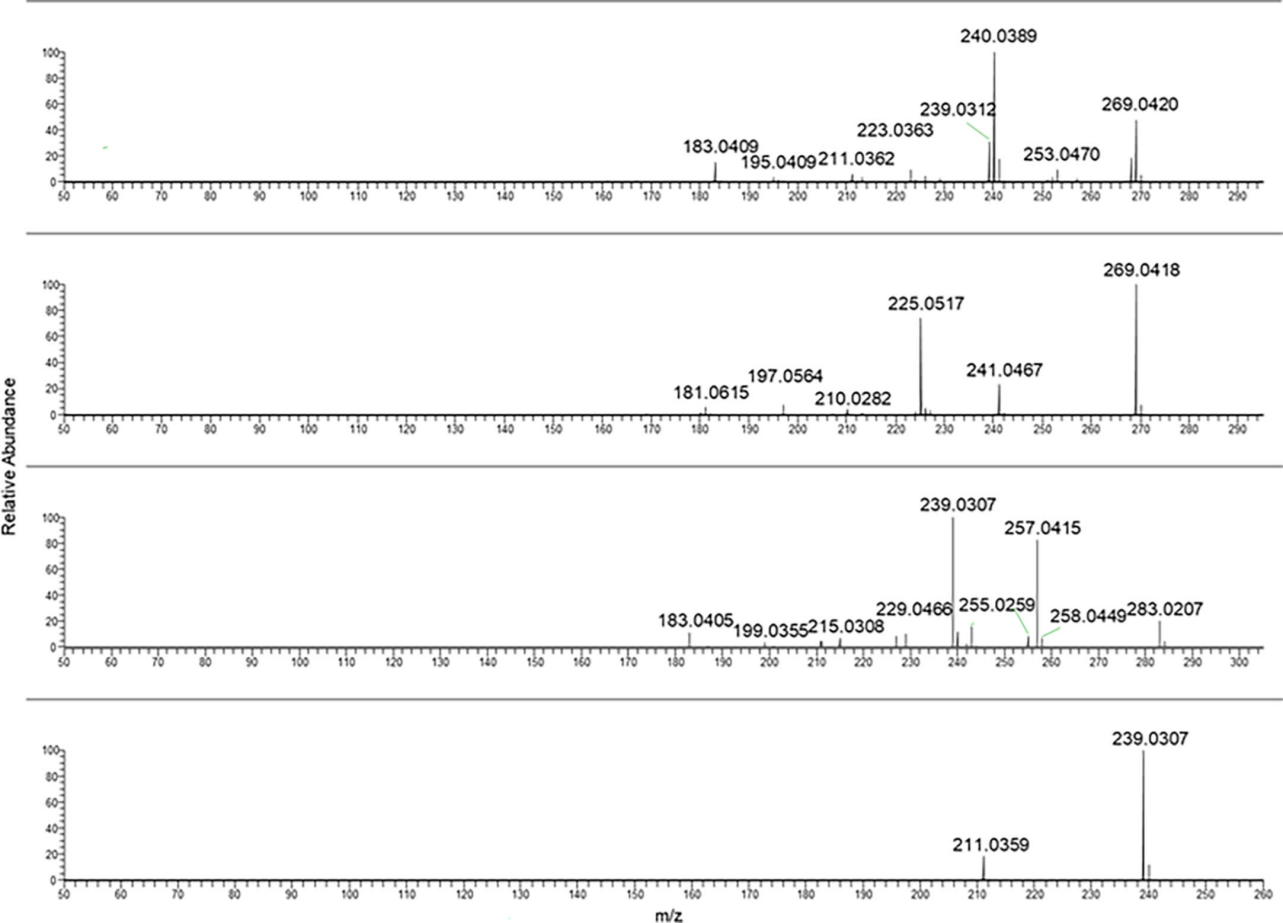
From the top to bottom: Aloe emodin, emodin, rhein and danthron MS/MS spectra.

**Figure 4:**
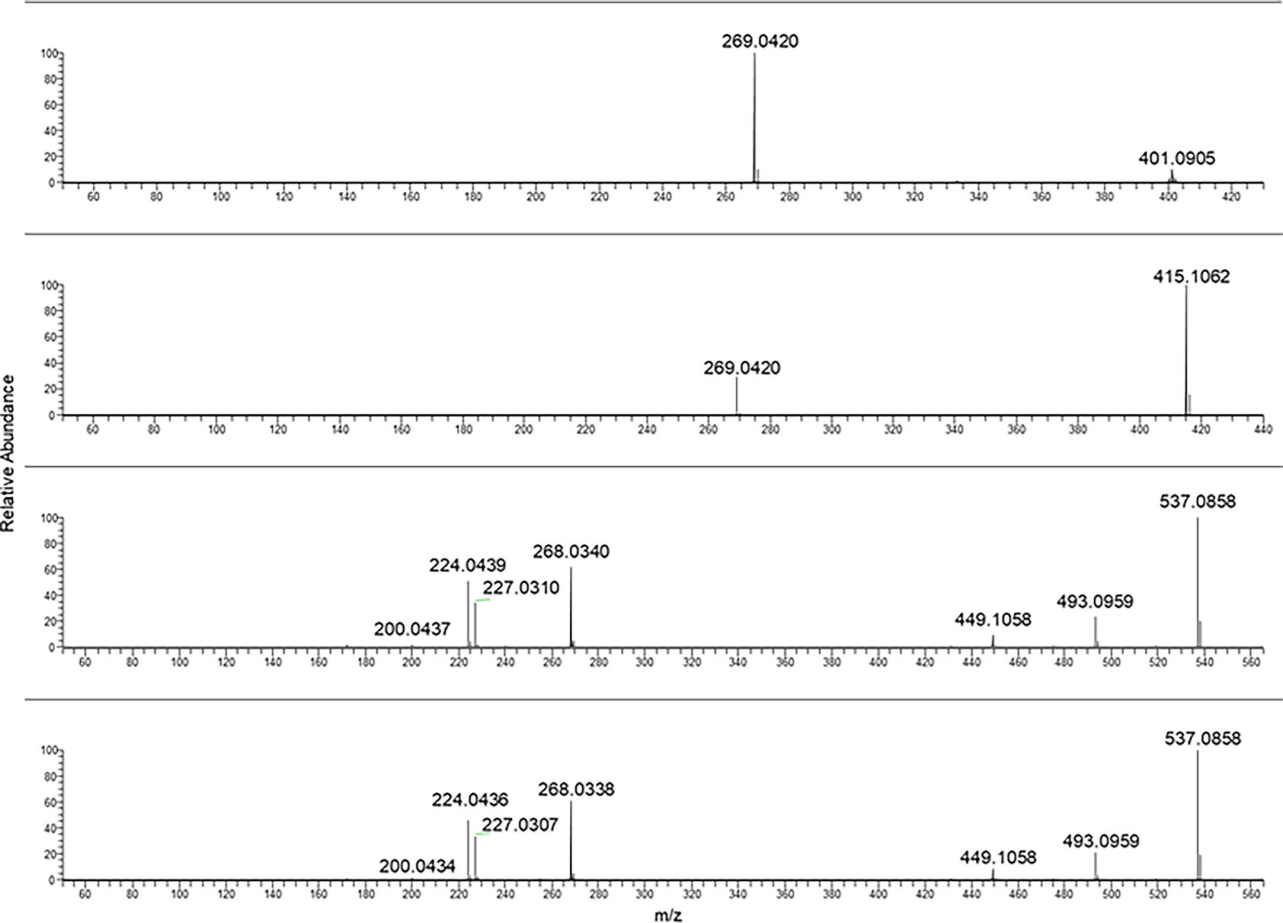
From the top to bottom: Frangulin B, frangulin A, sennidin B and A MS/MS spectra.

**Figure 5:**
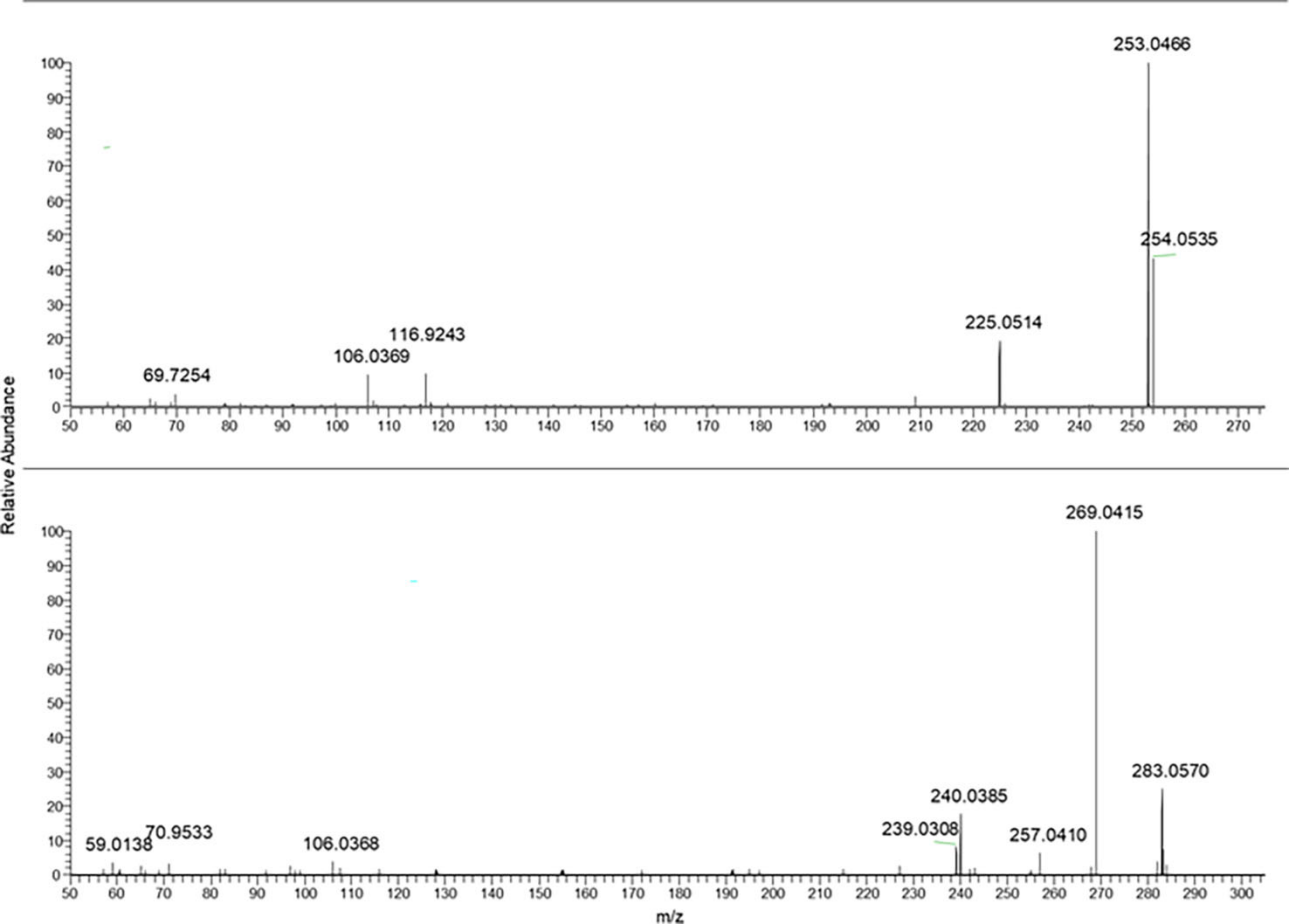
From the top to bottom: Crysophanol and physcion MS/MS spectra.

One of the aims of this work was to explore the presence and the relative ratio between different HAD. For this purpose, commercially available standards were recovered and used to setup a targeted LC-MS/MS method for identification and quantification of those analytes in the samples reported in **Table 1**. The first step was to optimize the ionization and fragmentation parameters. Each compound was dissolved and appropriately diluted for flow injection analysis. Both positive and negative ionization modes were explored for all, concluding that the negative ionization gave the best sensitivity for all the analytes. Different collision energies were tested for each one of the standard compounds to optimize the MS/MS fragmentation. As expected, the aglycone forms needed a higher collision energy to produce fragmentations. In **Figures 1**–**5**, the MS/MS spectra of the analyzed compounds were reported. The MS/MS spectrum of cascaroside A, obtained selecting the pseudomolecular ion [M-H]^−^ at *m/z* 579.17, was characterized by an intense fragment ion at *m/z* 459.13 (**Figure 1**). Sennoside A and B, see the second and the fourth MS/MS spectrum starting from the top of **Figure 1**, showed a fragment ion at *m/z* 699.13, originating from the [M-H]^−^ at *m/z* 861.19. The latter two are very close due to the isomeric structure of the two molecules.

The fragmentation of rhein-8-glucoside rising from the selection and collision of [M-H]^−^ at *m/z* 445.08 was characterized by a fragment ion at *m/z* 283.02.

In **Figure 2**, the characteristic fragmentations of aloin A and B as well as glucofrangulin A and B were showed.

Both the aloins and the glucofrangulins were structural isomers and showed very closed MS/MS spectra.

Selecting the pseudomolecular ion [M-H]^−^ at *m/z* 417.12, an intense fragment ion at *m/z* 297.07 was observed. The [M-H]^−^ at *m/z* 563.14 and at *m/z* 577.14 were selected for glucofrangulin B and glucofrangulin A and fragment ions at *m/z* 431.10 and at *m/z* 415.10, respectively.

Aloe-emodin and emodin MS/MS mass spectra were reported in **Figure 3** (two spectra from the top). As can be noticed, they showed the same chemical formula but different fragmentation.

In the case of rhein, the ionic species at *m/z* 283.02 was selected as pseudomolecular ion [M-H]^−^. The resulting MS/MS spectra were characterized by intense fragment ions at *m/z* 239.03 and 257.04.

In the case of frangulin B and frangulin A, the pseudomolecular ions [M-H]^−^ at *m/z* 401.09 and 415.10 were selected, and, as shown in **Figure 4**, they produced the same fragment ion at *m/z* 269.04.

Sennidin B and A yielded a superimposable MS/MS spectrum.

The fragmentation mass spectra of the last two analytes are reported in **Figure 5**.

Because some of the compounds were structural isomers and, as stated above, they produced very close MS/MS spectra, it was necessary to optimize the elution gradient to separate them as long as possible, without exceeding 30 min of the run time.

In **Table 2**, a summary of parameters for the anthraquinones considered for this study is reported. In particular, the chemical formula, the selected ions [M-H]^−^ used for the PRM analyses, the main fragment ion considered for the quantification, chosen from the MS/MS mass spectra as shown in **Figures 1**–**5**, the collision energy for the MS/MS spectra generation, and the retention time for each one were reported.

**Table 2:** List of analytes considered for the characterization of the samples listed in **Table 1**.

Compound	Formula	*m/z* [M-H]^−^	Fragment ion	HCD	RT (min)
Cascaroside A	C_27_H_32_O_14_	579,1719	459,132	20	2.18
Sennoside B	C_42_H_38_O_20_	861,1884	699,138	20	3.13
8-Glucorhein	C_21_H_18_O_11_	445,0776	283,022	10	6.45
Sennoside A	C_42_H_38_O_20_	861,1884	283,022	20	7.14
Aloin B	C_21_H_22_O_9_	417,1191	297,073	15	9.21
Aloin A	C_21_H_22_O_9_	417,1191	297,073	15	9.89
Glucofrangulin B	C_26_H_28_O_14_	563,1406	431,106	10	10.63
Glucofrangulin A	C_27_H_30_O_14_	577,1563	415,106	10	10.71
Aloe emodin	C_15_H_10_O_5_	269,0456	240,038	55	15.70
Frangulin B	C_20_H_18_O_9_	401,0878	269,042	10	16.08
Rhein	C_15_H_8_O_6_	283,0248	257,041	35	16.12
Frangulin A	C_21_H_20_O_9_	415,1035	269,042	10	16.17
Sennidin B	C_30_H_18_O_10_	537,0827	224,044	20	16.44
Sennidin A	C_30_H_18_O_10_	537,0827	224,044	20	17.24
Emodin	C_15_H_10_O_5_	269,0456	241,047	55	17.51
Danthron	C_14_H_8_O_4_	239,0350	211,036	45	18.03
Crysophanol	C_15_H_10_O_4_	253,0506	225,052	45	18.96
Physcion	C_16_H_12_O_5_	283,0612	269,042	35	19.44

### 3.2 Multivariate metabolomics data analysis

Metabolite profiling of 18 HADs derived from four different plants (*Cassia angustifolia*, *Rhamnus frangula*, *Rhamnus purshiana*, and *Rheum palmatum* and *raponticum*) was subjected to multivariate statistical analysis to understand the clustering and correlations between the investigated features and samples.

**Figure 6:**
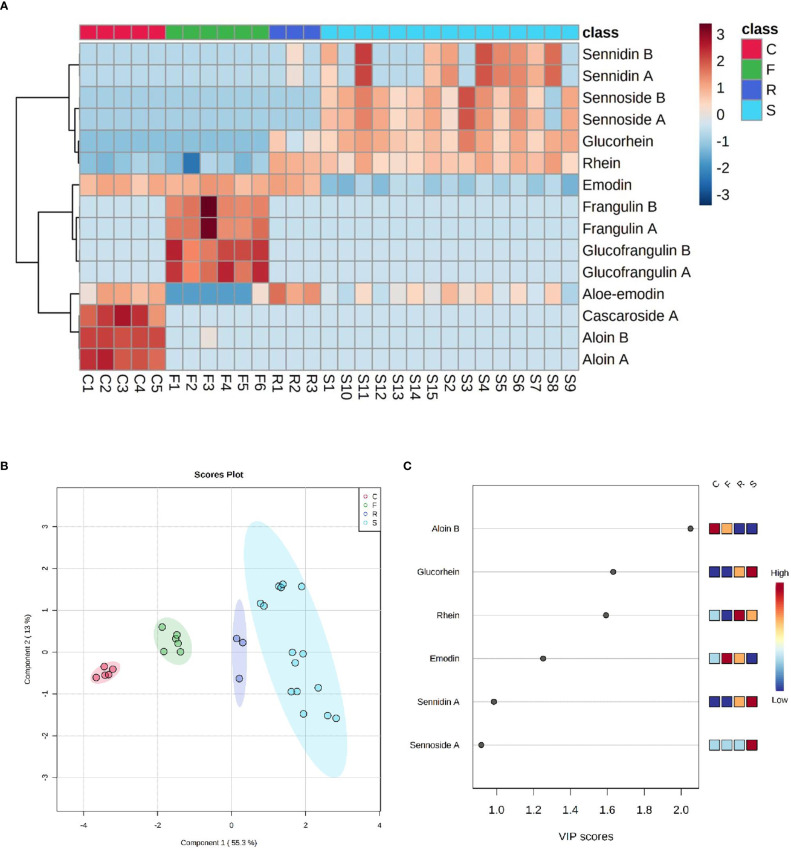
**(A)** Heatmap of HAD matrix, every row indicates a compound, every column a sample (C, Cascara - *Rhamnus purshiana*; F, Frangula - *Rhamnus frangula*; R, Rhubarb - *Rheum palmatum*; S, Senna - *Cassia angustifolia*). Red indicates high abundance, and light blue low abundance. **(B)** PLS-DA with four clusters of samples in different colors: red for C (Cascara - *Rhamnus purshiana*), green for F (Frangula - *Rhamnus frangula*), blue for R (Rhubarb - *Rheum palmatum*), and light-blue for S (Senna - *Cassia angustifolia*). **(C)** VIP score plot with the list of the most important metabolites allowing discrimination between samples (C, Cascara - *Rhamnus purshiana*; F, Frangula - *Rhamnus frangula*; R, Rhubarb - *Rheum palmatum;* S, Senna - *Cassia angustifolia*).

One of the 18 HADs is danthron, a synthetic and non-naturally occurring molecule, which has never been found in any sample and was, therefore, not reported in the statistical analysis. Like danthron, both the naturally occurring anthraquinones physcion and chrysophanol were never detected in the present analysis. In **Figure 6A**, an explorative overview of the chemical composition was reported to visualize the most representative HAD of each sample. Despite the variability of the samples and the different parts of plant employed, a common trend for each species was noticeable. In fact, the main components of *Rheum palmatum* resulted to be rhein, aloe-emodin, rhein-8-glucoside, and emodin. This last one, along with aloe-emodin, to a lesser extent, was also found in *Rhamnus purshiana* although cascaroside A, aloin A, and aloin B were the most characterizing metabolites of this botanical species. As far as *Rhamnus frangula* is concerned, both frangulins and glucofrangulins A and B were widely represented, followed by emodin, which was particularly low in *Cassia angustifolia*. This last plant is characterized by elevated levels not only of sennidin (both A and B) and sennoside (A and B) but also of rhein-8-glucoside and rhein. Although rhein-8-glucoside and rhein were present in all analyzed samples derived from *Cassia angustifolia*, it was noted that sennidin A and B showed a less constant trend. To investigate the common patterns among the different HAD, an HCA was used as a complimentary data reduction method for finding the underlying structure of objects through a repetitive process. The HCA dendrogram showed two main clusters. The first one included sennidin A and B, sennoside A and B, rhein-8-glucoside, and rhein; the second one consisted of all the others HADs, in turn, subdivided into two other subclusters. One of them involved glucofrangulins and frangulins, the *Rhamnus frangula*–characterizing compounds, and the emodin, which was also present in *Rhamnus purshiana* and *Rheum palmatum.* The other one included cascaroside A, aloin A, and aloin B, the *Rhamnus purshiana*–characterizing compounds, and the aloe-emodin, which was also found in all the other samples. In addition, supervised forms of discriminant analysis such as PLS-DA (Powell et al., 1996), which rely on the class membership of each observation, were also commonly applied in metabolic fingerprinting experiments (Powell et al., 1996). To identify the most important metabolites allowing discrimination between samples, a VIP values analysis was performed. Metabolites with a VIP value of >1.0 were considered as significantly discriminant. The findings suggested that the aloin B and emodin resulted to be the most discriminant compounds in *Rhamnus purshiana* and in *Rhamnus frangula*, respectively, whereas rhein-8-glucoside, sennidin A, and sennoside A in *Cassia angustifolia* (**Figures 6B, C**).

### 3.3 Citotoxicity assay

**Figure 7:**
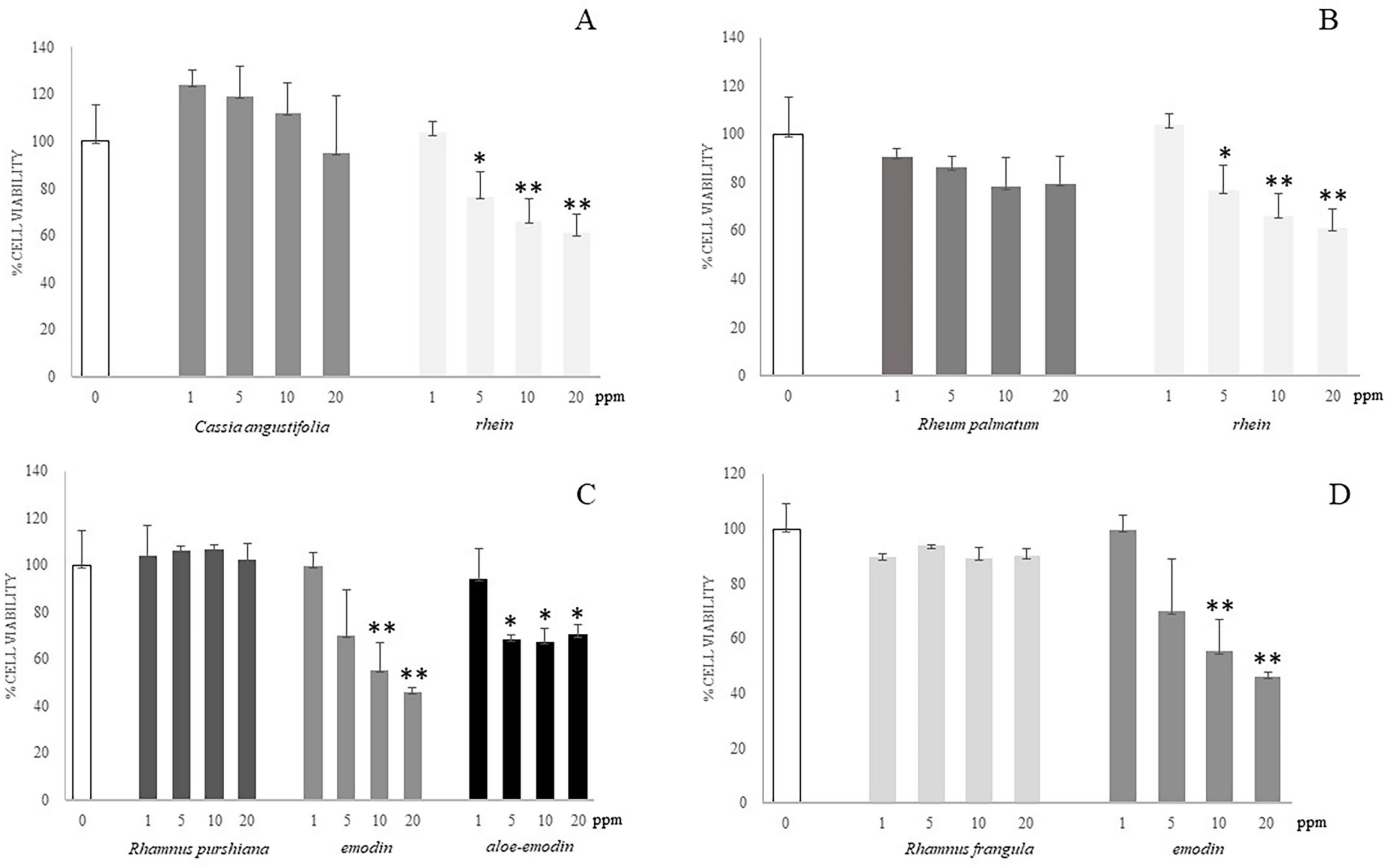
Cell viability of Caco-2 cells at different concentrations of different plant extracts and isolated compounds by alamarBlue viability assay. **(A)** Caco-2 cells were incubated for 48 h with different concentrations (1–20 ppm) of single molecule (rhein) or plant extract (*Cassia angustifolia*) at the same concentrations (1–20 ppm). **(B)** Caco-2 cells were incubated for 48 h with different concentrations (1–20 ppm) of single molecule (rhein) or plant extract (*Rheum palmatum*) at the same concentrations (1–20 ppm). **(C)** Caco-2 cells were incubated for 48 h with different concentrations (1–20 ppm) of single molecules (emodin and aloe-emodin) or plant extract (*Rhamnus purshiana*) at the same concentrations (1–20 ppm). **(D)** Caco-2 cells were incubated for 48 h with different concentrations (1–20 ppm) of single molecule (emodin) or plant extract (*Rhamnus frangula*) at the same concentrations (1–20 ppm). *P ≤ 0.05; **P ≤ 0.01.

To evaluate the contribution of each HAD compound and each whole plant extract to the toxicity of Caco-2 cells, we performed *in vitro* studies in which cells were exposed to different concentrations (1–20 ppm) of the single compounds (emodin, rhein, and aloe-emodin) and the crude extracts of different plants (*Cassia angustifolia*, *Rhamnus purshiana*, *Rhamnus frangula*, and *Rheum palmatum*) containing the same concentrations (1–20 ppm) of the abovementioned analytes. Moreover, the Caco-2 cells were treated also with the vehicle of 60% methanol in parallel with the abovementioned treatments for 48 h. First, we compared the whole extract of *Cassia angustifolia* with the single molecule of rhein because, from our analytical data, it resulted the most concentrated HAD in this plant extract. Data showed that the viability of Caco-2 treated with *Cassia angustifolia* at any concentrations did not undergo significant changes in comparison to control (**Figure 7A**). On the contrary, the treatment with rhein demonstrated an increasing of toxicity in Caco-2 cells: at 5 ppm, 76% cell viability vs. control (p < 0.05), showing a 66% of cell viability vs. control at 10 ppm (p < 0.01) and a 61% at 20 ppm (p < 0.01), indicating a dose-response manner (**Figure 7A**). The same kind of comparison was conducted using rhein, as single molecule, versus the whole extract of *Rheum Palmatum*, with rhein being its main HAD component. As reported in **Figure 7B**, the extract of *Rheum Palmatum* did not show any toxic events on Caco-2 viability at any concentrations.

As shown from the analytical data, the most concentrated HAD compounds in the *Rhamnus purshiana* extracts were resulted to be emodin and aloe-emodin; hence, we performed a comparison using the treatments with whole plant extract and these two single molecules at different concentrations (1–20 ppm). Emodin demonstrated a significant toxicity exhibiting a dose-dependent pattern with a reduction of cell viability of about 50% for the 10 ppm treatment (55% cell viability vs. control, p < 0.01) and a more evident decrease using the 20 ppm concentration (46% cell viability vs. control, p < 0.01) (**Figure 7C**). Aloe-emodin showed a significant reduction of viability after Caco-2 treatment with 5, 10, and 20 ppm (69%, 67%, and 70% vs. control, respectively, p < 0.05), without an evident correlation between dose-response. The treatment with vehicle did not show any toxicity in all the experiments (data not shown).

On the contrary, the treatments performed with *Rhamnus purshiana* extracts did not show any significant modifications of cell viability in comparison to control cells. Then, we performed the comparison between *Rhamnus frangula* extract and emodin (the most concentrated HAD resulting in this plant). Whereas emodin resulted to be toxic in a Caco-2 cell culture model, no significant toxicity resulted after treatment with *Rhamnus frangula* extract at any concentrations (**Figure 7D**).

Thus, the phytocomplexes did not show any significant modifications of cell viability in comparison to control cells, whereas the single-analyte treatment, at the same concentrations, induced cell toxicity.

### 3.4 Proteomics analysis

To further investigate the effect of HAD used as single molecule in comparison with the whole plant extracts containing HAD, we performed a label-free shotgun proteomic analysis to gain a comprehensive description of changes in protein expression that occur in Caco-2 cells. This approach allowed to better investigate the metabolic targeted or signaling pathways differentially involved in the Caco-2 samples treated with *Cassia angustifolia* and the single-molecule rhein. Analogously, a similar experiment was performed with *Rhamnus purshiana* and its most characterizing HAD, emodin. In particular, 3,279 quantifiable unique proteins with association to a known *Homo sapiens* gene were found to be in common in samples treated with rhein and *Cassia angustifolia*, whereas 452 and 323 were unique proteins derived by the treatment with single molecule and the phytocomplex, respectively. The “overlap index” based on Jaccard similarity coefficient (which measures similarity between finite sample subsets) was 0.80. For Caco-2 cell samples treated with rhein, 12.15% of the total proteome is unique, whereas 87.85% was in common with *Cassia angustifolia–*treated samples, characterized by 8.97% unique proteins. Similarly, for Caco-2 cell samples treated with emodin, 4.95% (118 proteins) of the total identified proteome was unique, whereas about 95% was in common with *Rhamnus purshiana* (Jaccard similarity coefficient of 0.77), characterized by 24.26% unique proteins (578 proteins).

Starting from the assumption deriving from application guidelines of regulation (EU) 468/2021, the HADs (like emodin and the structural analogs) have shown to be genotoxic *in vitro*, and the analysis was addressed to specifically investigate DNA damage and repair, apoptosis, and proliferation pathways associated with the use of whole plant extracts or the single molecule. Proteomic results showed that, in whole plant extract–treated samples, proteins involved in cell proliferation and negative regulation of the apoptotic process were identified. Conversely, in samples treated with the single molecule of HAD (rhein or emodin), proteins involved in apoptotic processes and in repairing DNA damage were found. In fact, exogenous stimuli like HAD could be responsible for increasing intracellular Reactive oxygen species (ROS), promoting DNA damage to induce mitochondrial dysfunction and subsequent mitochondrial mediated apoptosis (McDonald et al., 2022). In particular, intrinsic apoptosis pathway is triggered by DNA and oxidative damage, which are linked with disruption of the mitochondrial membrane potential (MMP), increase of cytochrome c (Cyt c) release, and apoptosis induction (McDonald et al., 2022). Precisely, from the comparison between rhein and *Cassia angustifolia*, several proteins involved in DNA damage repair were uniquely identified in rhein-treated samples, suggesting a potential genotoxic chemical exposure (Lam, 2022). These are the following:

- BRCA1-A complex subunit RAP80 (UIMC1) plays an important role in the DNA damage response by its localization at sites of DNA damage at double-strand breaks (Wu et al., 2012).
- Activating signal cointegrator 1 complex subunit 2 (ASCC2) plays a role in DNA damage repair as component of the ASCC complex by recruiting ASCC3 to sites of DNA damage (Jia et al., 2020).
- DNA ligases 1 (LIG1) is a critical enzyme of DNA metabolism, pivotal in DNA replication and in DNA repair pathways that require the re-synthesis of DNA (Timson et al., 2000).
- Tousled-like kinase 2 is an important regulator of recovery after DNA damage and cell cycle arrest in G2 (Bruinsma et al., 2016).

DNA damage and oxidative stress, which are linked with disruption of the MMP, increase of Cyt c release, and apoptosis induction, trigger intrinsic apoptotic pathway (McDonald et al., 2022).

In fact, in rhein unique subset, proteins involved in stimulating intrinsic apoptosis were detected and briefly described below.

- Transcriptional regulator QRICH1 is responsible for intrinsic apoptotic signaling pathway in response to endoplasmic reticulum stress (You et al., 2021). Intrinsic or mitochondrial apoptotic pathway is generally induced by DNA damage and caspase activation (Lopez and Bouchier-Hayes, 2022).

•Bcl-2–modifying factor positively induces apoptosis process, and it is involved in positive regulation of release of Cyt c from mitochondria (Hausmann et al., 2011). In fact, DNA damage activates the intrinsic pathway, leading to the activation of BH3-only proteins and the release of Cyt c from the mitochondria (Lopez and Bouchier-Hayes, 2022).

•Next to the previously described proteins involved in intrinsic apoptosis pathway, the fibrinogen alpha chain is involved in a negative regulation of extrinsic apoptosis pathway, confirming a preferential intrinsic apoptotic process in rhein-treated samples.

Differently, in *Cassia angustifolia*–treated samples, the main represented proteins were involved in a negative regulation of apoptotic pathways, such as interleukin-6 receptor subunit beta (Fischer and Hilfiker-Kleiner, 2007), and in cell proliferation process like protein regulator of cytokinesis 1 acting an important role also in apoptosis inhibition (Liu et al., 2018).

Similarly, an analogous result was also obtained from the comparison between emodin and *Rhamnus purshiana.* Our results confirm that the activity of emodin in increasing intracellular ROS and in promoting DNA damage (McDonald et al., 2022) by the identification of several proteins involved DNA damage repair process uniquely identified in emodin-treated samples. DNA ligases 1 (LIG1) was found also in this subset, along with ubiquitin carboxyl-terminal hydrolase 10 (UBP10) involved in autophagy and in the cascade of processes induced by the cell cycle regulator phosphoprotein p53, in response to the detection of DNA damage (Yuan et al., 2010).

Moreover, apoptotic proteins were identified in emodin subset:

- HCLS1-binding protein 3 induces cell apoptosis and activates AP-1 (Shi et al., 2011).
- PRKC apoptosis WT1 regulator protein is a pro-apoptotic protein capable of selectively inducing apoptosis by downregulating the anti-apoptotic protein BCL2 (Chakraborty et al., 2001).

No distinction between extrinsic and intrinsic apoptosis pathways has been detected in emodin samples.

*Rhamnus purshiana* unique protein subset showed, in a similar way to the *Cassia angustifolia*, proteins mainly involved in negative regulation of apoptotic pathways and cell proliferation process such as follows:

- Epidermal growth factor receptor is one of the most important signaling pathways that regulate growth, survival, proliferation, and differentiation in mammalian cells (Oda et al., 2005).
- Histone deacetylase 2 is involved in a positive cellular population proliferation (Weichert et al., 2008).

**Figure 8:**
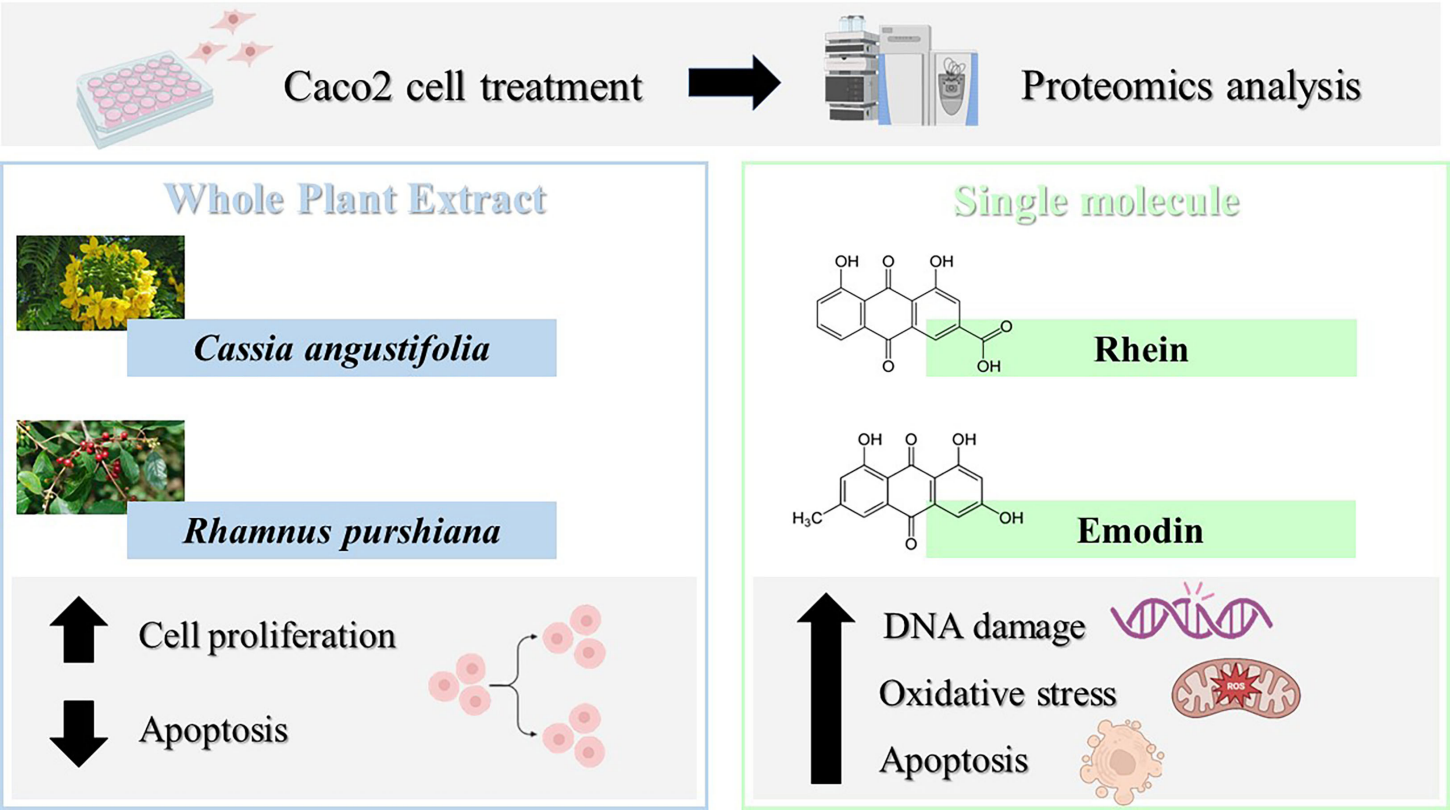
Caco-2 cell proteomics with different treatments (single molecule and phytocomplex) leads to the activation of different biological processes.

From this proteomic analysis, therefore, it emerges how single-molecule and phytocomplex treatment leads to the activation of different biological processes summarized in **Figure 8**.

## 4 Discussion

Over the years, there has been a worldwide interest in plants containing HAD (such as *Cassia angustifolia*, *Rhamnus purshiana*, *Rhamnus frangula*, *Rheum palmatum*, and *Rheum raponticum*) and isolated HAD due to their properties in supporting the regular bowel function. More than 700 different natural HADs have been found out distributed in roots, fruits, leaves, rhizomes, and flowers. Recently, the use of botanical products containing HAD (emodin, aloe-emodin, and danthron) has been re-evaluated by the European Union. Because there is the possibility of adverse health events associated with the use in foods of *Rheum*, *Cassia*, and *Rhamnus* and their preparations, but scientific uncertainty persists regarding the possible presence in these preparations of the substances listed in Annex III, Part A, of Regulation (EC) No. 1925/2006, these substances should be subject to Union surveillance and, therefore, be included in Annex III, Part C, of Regulation (EC) No. 1925/2006.

Therefore, during last 2 years, an increasing demand to perform monitoring of HAD content in commercial products has been occurred. To date, several published methods for detection and quantification of HAD were reported but they can perform a measurement of at least 10 HADs in the same analysis. Here, we set up a method that can detect and quantify 18 HAD molecules in a single run. In this study, an analytical method was set up using LC coupled to a high-resolution mass spectrometer to profiling anthraquinones levels in commercially available products.

The obtained results were subjected to a bioinformatic multivariate statistical assessment that allowed to achieve a general overview pivotal to understand the HAD composition samples by performing clustering and correlations analysis.

Despite the complexity of the analyzed samples, their natural variability, and their origin from different plant parts (e.g., leaves and bark), it was possible to confirm a specific trend of metabolites whose levels allow clustering among different plant species. For instance, the main components of *Rheum palmatum* resulted to be rhein, aloe-emodin, rhein-8-glucoside, and emodin; for *Rhamnus purshiana*, cascaroside A, aloin A, and aloin B were the most characterizing metabolites; for *Rhamnus frangula*, both frangulins and glucofrangulins A and B were widely represented, followed by emodin, which was conversely particularly low in *Cassia angustifolia*, mainly characterized by elevated levels of sennidin (both A and B) and sennoside (A and B), rhein-8-glucoside, and rhein. From the analysis, it was noticeable that aloin B and emodin resulted to be the most discriminant compounds in *Rhamnus purshiana* and in *Rhamnus frangula*, respectively, whereas rhein-8-glucoside, sennidin A, and sennoside A in *Cassia angustifolia.*

Once the anthraquinone composition of plant samples is analyzed, the second aim of this study is to evaluate the differential contribution of each HAD compound and each whole plant extract to the citotoxicity by performing *in vitro* studies on Caco-2 cells. Caco-2 were exposed to different concentrations (1–20 ppm) of the single compounds (emodin, rhein, and aloe-emodin) and the crude extracts of different plants (*Cassia angustifolia*, *Rhamnus purshiana*, *Rhamnus frangula*, and *Rheum palmatum*) containing the same concentrations (1–20 ppm) of the abovementioned analytes. The treatment with all the phytocomplexes did not show any significant modifications of cell viability in comparison to control cells differently from the single-analyte treatment at the same concentrations. Thus, the single-HAD treatment showed an increasing toxicity in Caco-2 cells. This is the reason why a deeper investigation was needed for the elucidation of molecular mechanisms underlying the cytotoxicity of the single molecule in comparison to the phytocomplex. In this context, we performed a label-free shotgun proteomic analysis to gain a comprehensive description of changes in expression of proteins involved in DNA repair, apoptosis, and proliferation pathways that occur in Caco-2 cells treated with *Cassia angustifolia* versus the single-molecule rhein and *Rhamnus purshiana* versus emodin, its most characterizing HAD. Proteomics analysis showed that HAD treatment could be compared to an exogenous stimulus implicated in increase of intracellular ROS, in promoting DNA damage to induce mitochondrial dysfunction and subsequent apoptosis. Specifically, from the comparison between rhein and *Cassia angustifolia*, several proteins involved in DNA damage repair and oxidative stress were uniquely identified in rhein-treated samples, suggesting a potential genotoxic chemical exposure that triggers an intrinsic apoptosis pathway concomitantly to a negative regulation of extrinsic apoptotic pathway. Indeed, a thought label-free proteomics and bioinformatic analysis was additionally possible to discriminate between the extrinsic and intrinsic pathways involved in apoptosis allowing to classify proteins as extrinsic or intrinsic signals. In particular, intrinsic apoptosis pathway is triggered by DNA and oxidative damage, which are linked with disruption of the MMP, increase of Cyt c release, and apoptosis induction (McDonald et al., 2022). Similarly, an analogous result was also obtained for emodin. In a previous work, it has been already confirmed that emodin increased intracellular ROS and promoted DNA damage to separately induce mitochondrial dysfunction and subsequent mitochondrial mediated apoptosis (McDonald et al., 2022). Our results confirm the involvement of several proteins in DNA damage repair process uniquely identified in emodin-treated samples. Moreover, apoptotic proteins were identified in emodin-treated cells, but, accordingly to the literature (Li et al., 2019), no distinction between extrinsic and intrinsic apoptosis pathways has been detected in emodin samples. This aspect confirmed a work of Li et al. (2019), in which how β-dihydroartemisinin-emodin (β-DHA-emodin) promotes apoptosis by activating both extrinsic and intrinsic pathways in human liver cancer cells was described (Li et al., 2019).

Conversely, in the whole plant extracts of *Rhamnus purshiana* and *Cassia angustifolia*, proteins were mainly involved in negative regulation of apoptotic pathways and cell proliferation process, suggesting a different molecular mechanism between the single-molecule and phytocomplex treatment.

## 5 Conclusion

Because some studies have reported that HADs (such as emodin and aloe-emodin) have shown genotoxic and carcinogenic activity, EFSA has recently re-evaluated the safety of medicinal plants containing such HAD in dietary supplements.

*In vitro* and *in vivo* studies concerning intestinal HAD toxicity have been mainly based on the use of the single molecules (specifically emodin, aloe-emodin, and rhein) so far but not on the evaluation of whole plant extract toxicity.

This aspect, along with the large variability in composition of HAD-containing products, led to the need to perform a phyochemical qualitative-quantitative characterization, which is the starting point of this study, aiming at assessing, for the first time, the toxic events of HAD used as single molecule in comparison with the whole plant extracts. First, cell viability was evaluated by the commonly used cytotoxicity assay that can elicit the preliminary HAD action, and then, an innovative shotgun proteomics approach coupled with bioinformatic analysis was applied to profile the differential proteome changes in response to performed treatment (single HAD versus whole plant extract).

## Data availability statement

The original contributions presented in the study are included in the article/**Supplementary Material**. Further inquiries can be directed to the corresponding author.

## Author contributions

LT, VC, LS, and PN contributed to the conception and design of the study, performed experiments, and wrote the manuscript. BB, RP, GI, and PE contributed to the conception of the study and regulatory support. All authors contributed to the article and approved the submitted version.
